# Sprint Variables Are Associated with the Odds Ratios of Non-Contact Injuries in Professional Soccer Players

**DOI:** 10.3390/ijerph181910417

**Published:** 2021-10-03

**Authors:** Hadi Nobari, Elena Mainer-Pardos, Angel Denche Zamorano, Thomas G. Bowman, Filipe Manuel Clemente, Jorge Pérez-Gómez

**Affiliations:** 1 Department of Physical Education and Sports, University of Granada, 18010 Granada, Spain; 2 HEME Research Group, Faculty of Sport Sciences, University of Extremadura, 10003 Cáceres, Spain; angeldenche@gmail.com (A.D.Z.); jorgepg100@gmail.com (J.P.-G.); 3 Department of Exercise Physiology, Faculty of Educational Sciences and Psychology, University of Mohaghegh Ardabili, Ardabil 56199-11367, Iran; 4 Sports Scientist, Sepahan Football Club, Isfahan 81887-78473, Iran; 5 Health Sciences Faculty, Universidad San Jorge, Autov A23 km 299, 50830 Villanueva de Gállego, Spain; 6 Department of Athletic Training, College of Health Sciences, University of Lynchburg, Lynchburg, VA 24501, USA; bowman.t@lynchburg.edu; 7 Escola Superior Desporto e Lazer, Instituto Politécnico de Viana do Castelo, Rua Escola Industrial e Comercial de Nun’Álvares, 4900-347 Viana do Castelo, Portugal; Filipe.clemente5@gmail.com; 8 Instituto de Telecomunicações, Delegação da Covilhã, 1049-001 Lisboa, Portugal

**Keywords:** football, injury risk, high load, external monitoring, performance, high-speed distance, global positioning system

## Abstract

Significant evidence has emerged that a high volume of sprinting during training is associated with an increased risk of non-contact injuries in professional soccer players. Training load has been reported as a modifiable risk factor for successive injury in soccer. Sprint workload measures and non-contact injuries were recorded weekly in twenty-one professional soccer players over a one season period. Odds ratio (OR) and relative risk (RR) were calculated based on the weeks of high and low load of total distance (TD), high-speed distance (HSD), sprint distance (SPD). and repeated sprints (RS). The Poisson distribution estimated the interval time between the last injury and the new injury. The weeks with high-load levels increased the risk of non-contact injury associated with TD (OR: 4.1; RR: 2.4), HSD (OR: 4.6; RR: 2.6), SPD (OR: 6.9; RR: 3.7), and RS (OR: 4.3; RR: 2.7). The time between injuries was significantly longer in weeks of low-load in TD (rate ratio time (RRT) 1.5 vs. 4.2), HSD (RRT: 1.6 vs. 4.6), and SPD (RRT: 1.7 vs. 7.7) compared to weeks of high-load. The findings highlight an increased risk of non-contact injuries during high weekly sprint workloads. Possibly, TD, HSD, and SPD measured via a wearable inertial measurement unit could be modeled to track training and to reduce non-contact injuries. Finally, the interval time between the last injury and the new injury at the high-load is shorter than the low-load.

## 1. Introduction

Soccer is considered an intermittent sport and it demands a wide variety of skills at high intensity with periods of rest or low intensity [1]. Professional soccer players have a congested calendar which usually requires playing successive matches with three days of recovery [2]. These players are exposed to high training load due to the poor recovery periods between years of long training and high match frequencies. These competitive demands could increase injury risk and reduce performance; therefore, they could be detrimental to team success [2,3,4]. 

In recent times, there has been an increase of high-speed distance (HSD) and running during competitive soccer matches [5]. Moreover, the ability to produce HSD seems to be a significant quality for performance [6]. Soccer players need improved development of high speed and sprint running ability to gain advantage in attacking and defensive situations [7]. Malone et al. [8] reported that exposing players to large and rapid increases in HSD and sprint distances (SD) increased the odds of injury. Bowen et al. [9] demonstrated that three weekly accumulations of accelerations >9254 were related to an elevated risk of non-contact injury. Nevertheless, from an injury perspective, more studies are warranted that allow coaches to understand the dose-response of HSD within training environments. 

Micro-technology use, such as global position systems (GPS) which measure external training loads of players, has become prevalent in professional soccer [10]. The tool is used to identify the activity profile of players during training and matches [10]. Also, GPS is able to quantify the distance covered along with high acceleration efforts, short duration, high velocity sprints and repeated sprint (RS) exercise bouts [11]. Angelidis et al. [12] examined the link between GPS variables and non-contact injuries in soccer players in a recent systematic review. They found eight variables, total distance (TD), high-speed running, total load, accelerations, decelerations, new body load, meter per minute, and sprinting, that deserve particular attention when monitoring soccer players’ external load for the purpose of injury prevention. At each training and competition, players are exposed to a given workload [13]. An inappropriate workload during these periods can increase injury risk. An increased acute GPS-derived workload in team sports produced a higher association with injury risk [14,15]. In professional soccer players, when the chronic loads were low with very high acute spikes, non-contact injury risk increased [16]. However, more studies are needed to consider the effect of the accumulation load to establish stronger conclusions between GPS training load measurement and non-contact injury in professional soccer players.

Time loss due to injury is one of the bigger problems during soccer players’ careers [3]. Most injuries occur in the lower extremities [17]. In addition, injuries of professional soccer players have a considerable impact on the sports industry [18], the recovery and rehabilitation of players is associated with a considerable cost [19]. A non-contact injury is an injury with no physical contact with other players, and a considerable proportion of injuries in soccer are non-contact [17]. Talukder et al. [20] proposed that the most relevant characteristics for predicting injuries are the average speed, the number of past competitions played, the average distance covered, the number of minutes played to date, and the average field goals attempted. Hence, researchers and coaches are interested in reducing the likelihood of injuries to their soccer players and consequently, injury forecasting is engaging more interest within the sporting environment.

Training load has been reported as a modifiable risk factor for successive injury in soccer [21]. Gabbet and Ullah [22] observed a correlation between high intensity running and injury risk during training sessions. Within elite soccer players, maintaining heart rate (HR) above 85% HRmax during training was associated with increased risk of injury [23]. Malone et al. [8] recently showed that higher chronic loads and higher aerobic conditioning appear to offer a better protective effect against injury for professional soccer players, and they should be considered mediators of injury risk. It is important for practitioners to understand the optimal training load at which adaptation occurs without raising the risk of injury. However, there are no studies that have investigated the relationship between non-contact injury incidence and sprint variables through GPS within professional soccer players. 

Given the need for coaches and practitioners to know the relationship between GPS-derived workload and non-contact injury risk in professional soccer players, the aims of the current study were to investigate, (i) the relationship between the total distance, HSD, sprint distance (SPD), and RS with non-contact injuries in professional players throughout a full soccer season; (ii) the injury risk associated between high- versus low-load level for each of the aforementioned parameters with odds ratios (OR) and relative risk (RR), respectively; and (iii) ultimately, a Poisson test to obtain lambda values (i.e., number of injuries per week for each level of cases listed), with the predicted time between injuries to new injury to calculate the rate ratio.

## 2. Materials and Methods

### 2.1. Participants

Twenty-one soccer players [age (year), 28.3 ± 3.8; height (cm), 181.2 ± 7.1; weight (kg), 74.5 ± 7.7] from a professional team in the Persian Gulf Pro League were analyzed during a full season (2018–2019 years). These players had more than 8 years of professional playing experience. The criterion for entering the participants’ information into the analysis was that they had participated in at least three training sessions per week. The criterion for excluding participants’ data in the analysis was that data was not available for 2 consecutive weeks or they had not participated for lest 2 consecutive weeks in the training. Also, goalkeepers’ data were eliminated in this study. Based on a power analysis of results from previous studies, we believed a sample size of 21 was adequate to reduce the risk of a Type 2 error [8,22]. This research was conducted by the training coaches of the club after approval with the relevant authorities and the head coach in the club. Prior to commencing the study, the approval of the research ethics committee from the University of Isfahan (IR.UI.REC.1399.064) was also received. All players were informed of the purpose of the study before signing the informed consent. All stages of this study were carried out according to the human studies in the Helsinki Declaration.

### 2.2. Study Design

The design of the prospective cohort study was performed in a full season during the Persian Gulf Pro League and knockout tournament. External load monitoring was performed by GPS (GPSPORTS systems Pty Ltd, Model: SPI High Performance Unit (HPU), Canberra, Australia) at each training and match session over the whole season. All non-contact injuries (that is, occurring without contact with foreign material or athletes) were recorded during the season. During the full season, 7-weeks congested play (i.e., two or more matches within 7-days), 30-weeks non-congested play; 44 matches, 11-weeks with no competitions, 200 training sessions, and 14,126.9 minutes of time play and sessions were held. Almost all training sessions and competitions were held on a natural grass field. The team between weeks 26 to 32 (mid-season break) was at the International Camp in Turkey. The running variables recorded, during the season, for this study included: TD, HSD (18–23 km/h^−1^) [24,25] SPD, and meters of RS. Afterward, each variable was divided into two levels, upper and lower, and subsequently, the relationship between the variables was measured. 

### 2.3. Procedure 

#### 2.3.1. Wearable Inertial Measurement Unit Receiver

All players’ activities in training sessions and matches were recorded with GPSPORTS systems Pty Ltd, Canberra, Australia. The GPS-based tracking systems for professional athletes, model SPI HPU features included: 15 Hz position GPS, distance, and speed measurement; accelerometer: 100 Hz, 16 G Tri-Axial-Track impacts, accelerations, and declarations as well as data source body load (BL); Mag: 50 Hz, Tri-Axial; dimensions: 74 mm × 42 mm × 16 mm; water resistance and data transmission: infra-red and weighed 56 g. Previous studies have shown that the GPS unit had very high accuracy and demonstrated excellent validity and inter-unit reliability [26]. All data were collected during training and match sessions with favorable weather and GPS satellite status.

#### 2.3.2. Data Collection

Data collection was completed as in previous studies [27,28]. As in during pre-session we placed upright tracking units in the pouch of the manufacturer provided belt, then ensured the green lights (GPS tracking) and red lights (heart-rate tracking) were flashing. Post-session, tracking units were collected from players and placed on the docking station. Data from the units were automatically downloaded then deleted from the units to prepare for the next session. After 10 min, the units turned off automatically. The GPS system was tuned to the default SPI IQ Absolutes in this study. 

#### 2.3.3. Sprint Variables Calculated

*TD* was calculated as the mean number of weekly meters run by the team’s players over the 48 weeks of the season.

*HSD* was calculated as the mean of weekly meters run at high speed by the team’s players over the 48 weeks of the season.

*SPD* was calculated as the mean number of weekly meters run by the team’s players during the 48 weeks of the season.

*RS* was calculated as the mean weekly meters of repeated sprints performed by the team’s players throughout the 48 weeks of the season.

*TD* level division was the difference between “high-load” and “low-load” weeks according to the average weekly TD of the team. “High-load” was defined as TD ≥ 21,900 and “low-load” was defined as TD < 21,900, both in meters. The cut-off point was established in the TD value, in which a greater difference was found between the mean of injuries between weeks of higher and lower load (clusters with more than 11 weeks), having previously ordered the weeks in accordance with higher to lower TD.

 *I.*  *Mean of injuries during the 12 weeks of highest TD–Mean of injuries during the rest of the weeks.*
 *II.*  *Mean of injuries during the 13 weeks of higher TD–Mean of injuries during the rest of the weeks.*
 *III.*  *Mean of injuries during the 36 weeks of greater TD–Mean of injuries during the rest of the weeks.*

*HSD* level division was the difference between “high-load” and “low-load” weeks according to the average weekly HSD of the team. “High-load” was defined as HSD  ≥288 and “low-load” was defined as HSD < 288, both in meters. The cut-off point was established at the HSD value in which a greater difference was found between the mean of injuries between weeks of higher and lower load (clusters with more than 11 weeks), having previously ordered the weeks in accordance with higher to lower HSD.

 *I.*  *Mean of injuries during the 11 weeks of highest HSD–Mean of injuries during the rest of the weeks.*
 *II.*  *Mean of injuries during the 12 weeks of higher HSD–Mean of injuries during the rest of the weeks.*
 *III.*  *Mean of injuries during the 36 weeks of higher HSD–Mean of injuries during the rest of the weeks.*

*SPD* level division was the difference between “high-load” and “low-load” weeks according to the average weekly SPD of the team. “High-load” was defined as SPD  ≥1601 and “low-load” was defined as SPD < 1601, both in meters. The cut-off point was established at the SPD value in which a greater difference was found between the mean of injuries between weeks of higher and lower load (clusters with more than 11 weeks), having previously ordered the weeks in accordance with higher to lower SPD.

 *I.*  *Mean of injuries during the 11 weeks of highest SPD–Mean of injuries during the rest of the weeks*.
 *II.*  *Mean of injuries during the 12 weeks of higher SPD–Mean of injuries during the rest of the weeks*.
 *III.*  *Mean of injuries during the 36 weeks of higher SPD–Mean of injuries during the rest of the weeks*.

*RS* level division was the difference between “high-load” and “low-load” weeks according to the average weekly RS of the team. “High-load” was defined as RS  ≥60 and “low-load” was defined as RS < 60, both in numbers. The cut-off point was established at the RS value in which a greater difference was found between the mean of injuries between weeks of higher and lower load (clusters with more than 11 weeks), having previously ordered the weeks in accordance with higher to lower RS.

 *I.*  *Mean of injuries during the 11 weeks of highest RS–Mean of injuries during the rest of the weeks.*
 *II.*  *Mean of injuries during the 12 weeks of higher RS–Mean of injuries during the rest of the weeks.*
 *III.*  *Mean of injuries during the 36 weeks of higher RS–Mean of injuries during the rest of the weeks.*

#### 2.3.4. How to Record and Calculate Injury

Information on injuries was updated daily by the team’s specialized medical staff. All injuries were recorded by type, location of the injury, and timing of injury based on previous studies. The information used for the injuries was as follows:

1. The number of registered injuries was the total number of non-contact injuries per week for the team over the 48 weeks of the season.
2. Weekly injury recorded the existence or not of a non-contact injury in each of the 48 weeks of the season.

### 2.4. Statistical Analysis

IBM SPSS 25 and R Studio 3.6.2 (Statistical Computing, Vienna, Austria). were used for the statistical analyses. A descriptive statistical analysis was performed, indicating the mean values and SD of the “high-load” and “low-load” levels for the variables “TD”, “HSD”, “SPD”, and “RS”, as well as the total values. Non-parametric Mann–Whitney U tests were used to compare the median of the load levels of the previous variables, checking the existence of statistically significant differences between them. A test of normality, Kolmogorov–Smirnov, verified that the variable “Number of injuries”, did not follow a normal distribution. Additionally, a descriptive analysis of the number of injuries produced in the weeks of high and low load of each one of the variables was completed, as well as the calculation of the means of each one of them, both for the two levels of load, as well as for the total. In order to detect statistically significant inter-group differences between the means of injuries at the “high-load” and “low-load” levels of the “TD”, “HSD”, “SPD”, and “RS” variables, non-parametric tests were used, considering, as factors, the load levels of each variable. A contrast of proportions determined if significant differences existed between the levels of “high-load” and “low-load” of each variable and the weeks with injury. To estimate the risk of having a high-load level compared to a low-load level, respectively, of each variable, OR and RR were calculated, in addition to the respective confidence intervals (CI) 95%. Finally, the variable “Number of injuries” followed a Poisson distribution, allowing the performance of a Poisson test, obtaining the lambda values (average number of injuries per week for each level of load for each variable), and the expected time until a new injury occurred, once one had occurred. Possible significant differences between load levels were examined via calculating Rate Ratios, as well as respective CI 95%. The level of significance was set at *p* < 0.05 and *p* < 0.001 for all stages.

## 3. Results

A descriptive analysis of the high- and low-load levels for the TD, HSD, SPD, and RS variables are presented in Table 1. Statistically significant differences between “high-” and “low-” load levels were observed in all variables.

Table 2 details the relation between sprint variables with non-contact injuries. Mean injuries were significantly higher in the high load weeks compared to the low load weeks for TD, HSD, SD, and RS.

Significant differences were found (*p* < 0.05) in the proportion of injury-free weeks between high- and low-load weeks in all variables studied. The OR and RR of producing some injury without contact was significantly higher in the weeks of high load compared to the weeks of low load in each one of the variables of interest. Similarly, significant RR were found for all variables except for the “RS” variable (Table 3).

Ultimately, it was observed that the time between injuries was reduced in weeks of high-load and longer in weeks of low-load in the four variables studied, although significant differences were found in all variables, except in the variable RS. Similarly, the rate ratios were significant in all variables for the expected weeks between injuries except for the variable RS (Table 4).

## 4. Discussion

This study investigated the relationship between GPS-derived workload and non-contact injuries in professional soccer players. The present data highlight that the weeks of high-load levels increase the risk of non-contact injury within soccer players. Based on our results, TD, HSD, and SPD variables could potentially track training and may allow exercise prescription to reduce non-contact injuries. Finally, according to the rate ratios calculated, the interval time between the last non-contact injury and the new injury during high-load training in TD, HSD, and SPD) is shorter than during low-load training. 

The relationship between non-contact injuries and different levels of load to find the expected time until a new non-contact injury was presented via ORs and RRs. All variables were significant except for the RS variable in RR (Table 3). TD and HSD were found previously to be the best rates of non-contact injuries [12]. In the current study and in previous studies, increased injury occurred when ORs and RRs were greater than 1 [12,29]. TD (OR: 4.1, RR: 2.4) and HSD (OR: 4.6, RR: 2.6) were significantly different between high- and low-load weeks. Therefore, participants had a higher odds and risk of a non-contact injury during high-load weeks compared to low-load weeks. Previous studies did not investigate SPD and RS, although these have been found to be associated with non-contact injury occurrence [12]. In the current study, when players covered SPD ≥ 1601 m during a week, they were at significantly higher risk of injury compared to weeks with SPD < 1601 m (OR: 6.9; RR: 3.7 m; *p* < 0.05). However, RS reported a significant OR (4.3; *p* < 0.05) but no significant RR (2.7; *p* > 0.05). Therefore, TD, HSD, and SPD variables could potentially be associated with non-contact injuries and the expected time until new injury and may help practitioners and coaches adjust training load in an effort to prevent non-contact injury.

Our study is one of the first to investigate the effects of GPS-derived workload on subsequent non-contact injury risk in an elite cohort of soccer players. We observed that the weeks in which players reached TD < 21,900 m, HSD < 288 m and SPD < 1601 m, they reduced injury risk compared to higher week loads (OR: 4.1 to 6.9). In addition, our findings suggest that weeks in which players repeated <60 sprints, they were at reduced injury risk compared to high-load weeks (OR: 4.3). The current data suggest that low load exposure to sprint variables can protect professional soccer players from subsequent non-contact injury risk. These results could provide coaches and practitioners with initial guidelines for optimal workload to reduce non-contact injury occurrence. 

Previous literature has found that moderate and higher chronic training loads can offer a protective effect against lower limb injury risk for team sport players [8,28]. Malone et al. [30] observed that players with moderate exposures to maximum velocity (>6–10) were at reduced injury risk compared to players who experienced lower (<5) exposures (OR: 0.24). Also, the authors [30] found that players with higher 21-day chronic loads (≥2584 AU) completed increased high-speed and sprint running distances that indicated a protective effect against injury (OR: 0.24–1.22). The reason for these observations may be because the players are usually exposed to a chronic training load period. Hence, they are used to tolerating high-speed running workload which reduces injury risk. In contrast, Malone et al. reported that elite soccer players were at increased risk of injury when they experienced high one-week cumulative training loads (≥1500 to ≤2120 AU) [8]. Gabbet and Ullah [22] demonstrated that greater amounts of HSD are associated with an increased injury risk of lower body soft-tissue injury in elite rugby players (RR: 2.7). These findings can be explained by higher acute loads that are associated with an increase in fatigue status in players and resultant increases in injury risk [21]. In this regard, controversy still exists regarding the impact of sprints in non-contact injuries and further studies are needed in the elite soccer environment.

From a performance point of view, careful thought should be taken to understand and apply the present findings to elite performance soccer. Our data suggest that reducing the amount of sprinting in professional soccer training could offer a protective effect against non-contact injury. In this context, a fine balance exists between training load restriction (i.e., preventing injury) and increased training loads (i.e., improving performance) [4,8,22]. Consequently, keeping in mind the need for an appropriate stimulus to improve performance, we used the current data to vary sprinting loads during the soccer season. Our data suggest that players will be exposed to increased risk of non-contact injury when the amount of TD, HSD, SPD, and number of RS is higher. Planning a decrease of mean sprint variables in some high-load weeks may offer the balance between injury prevention and performance enhancement. The finding could be an important consideration to correctly manipulate workloads during the season, not only to reduce non-contact injury but also to enhance physical performance within professional soccer players. 

A method of measuring internal training load, such as rating of perceived exertion (RPE) using the category ratio scale (CR10-scale) per session [31], could be used to assess how the players are coping with training loads every day. This method has been shown to be the most valid indicator of exercise intensity [32]. The collection of weekly GPS (external load) and session-RPE (internal load) variables allows the calculation of chronic training loads [2], enabling reduction of future loads based on these variables. Previously, Gallo et al. [33] demonstrated moderate to very large associations between session-RPE and both HSD (r = 0.51) and TD (r = 0.81) in team sport athletes. Therefore, including this parameter in future research could assist practitioners and coaches in finding the appropriate stimulus and balance between injuries and performance.

Recent studies observed that non-contact injuries had a high frequency of re-injury and persistent complaints after return to soccer competition [3,34]. According to the results of the rate ratios, the interval time between the last non-contact injury and the new injury at the high-load in TD, HSD, and SPD is shorter than the low-load (Table 4). Soccer injuries are defined by an inter-individual variability of flexor and extensor muscle performances [35]. One of the major risk factors that has been identified is strength asymmetry, and the asymmetry index has been considered as a valid and useful tool to detect players at high injury risk (e.g., 4-fold in players with >10% asymmetry) of lower extremity injury [36]. Strength asymmetry has been considered in relation to movement speed in team-sport athletes [37]. Therefore, the major number of sprints during high load weeks may induce an increase of strength imbalances in the lower limbs, and a greater predisposition to injury may be possible. Consequently, inter-limb asymmetry tests and strength training programs may allow a preventive approach to reduce the risk of re-injury in professional soccer.

### 4.1. Limitations

Several limitations are important to mention from the current study. Injury risk in soccer players can also be attributed to multiple factors such as previous injury, perceived muscle fatigue, nutrition and hydration, mood, sleep ratings, and physiological stressors, none of which were included in this study [18,27]. Research incorporating objective measures of GPS with RPE-values may provide additional insight into the training load–injury relationship. In addition, the present investigation was developed in one professional soccer team during one season. Consequently, our results cannot be directly extrapolated to other sports, teams, or across multiple seasons. Therefore, we recommend further studies for better understanding of the workload–injury relationship in elite soccer. 

### 4.2. Practical Applications

The current study is the first to provide an indication of how players’ weekly training load is associated with non-contact injuries in professional soccer players. Team soccer staff should measure weekly the internal and external load of players to plan and implement optimal trainings that improve their performance and reduce their injury risk. Given that these findings suggest that a high load of sprints increases the risk of sustaining non-contact injuries, attempting to adjust training load for sprint variables during high load weeks is recommended. However, this observation requires further investigations. One possibility to achieve this in practice is to restrict the amount of sprinting and prescribe stable and consistent weekly loads during the season to prevent any spikes in acute workload. Our results also suggest that TD, HSD, and SPD measured via GPS could be modelled to track training and to reduce non-contact injuries. Nevertheless, further studies are recommended to improve the accuracy of these variables in reducing non-contact injuries. Finally, the findings may promote an evidence-based approach for coaches and practitioners in planning and monitoring weekly training load thresholds to reduce fatigue during soccer participation and consequently, prevent non-contact injuries.

## 5. Conclusions

The results of this study demonstrated that the high volume of sprinting during high-load weeks is associated with non-contact injury occurrence. In addition, TD, HSD, and SPD of GPS variables could track training and may allow exercise prescription to reduce non-contact injuries. Finally, the interval time between the previous injury and a new injury during high load training is shorter than during low load training. The current data provide an important contribution that may be valuable to support the decisions of coaches and practitioners when they have to choose the best GPS variables to observe non-contact injuries, to reduce the amount of sprinting during professional soccer seasons, and to adjust training loads for sprint variables. However, they could also contemplate the consequences of reducing training loads during the season on playing performance.

## Figures and Tables

**Table 1:** Descriptive information of sprint variables based on high- and low-load levels.

Variables	High-Load Median (IQR)	Low-Load Median (IQR)	*p*	Total Median (IQR)
TD (m)	23,757 (21,073–26,367)	20,006 (21,667–25,828)	≤0.001 ***	21,595 (19,211–25,046)
HSD (m)	360.2 (289.5–387.8)	207.7 (157.9–235.2)	≤0.001 ***	288.2 (210.4–361.7)
SPD (m)	1962 (1854–2473)	1429 (1189–1590)	≤0.001 ***	1845 (1518–2182)
RS (m)	77.6 (65.1–85.2)	48.6 (37.9–57.6)	≤0.001 ***	65.5 (55.9–81.0)

IQR: Interquartile range; m: meters; Total distance (TD): High load (weeks with TD ≥ 21,900 m); Low load (weeks with TD < 21,900 m); High-speed distance (HSD): High load (weeks with HSD ≥ 288 m); Low load (weeks with HSD < 288 m); Sprint distance (SPD): High load (weeks with SPD ≥ 1601 m); Low load (weeks with SPD < 1601 m); Repeated sprints (RS): High load (RS ≥ 60). Low load (RS < 60); *p* (*p* value); *** (*p* < 0.001).

**Table 2:** Relation between TD, HSD, SPD, and RS with non-contact injuries (mean).

Variables	High-Load			Low-Load			*p*	Total		
	Injuries	Weeks	M	Injuries	Weeks	M		Injuries	Weeks	M
TD (m)	15	23	0.65	6	25	0.24	0.017 *	21	48	0.44
HSD (m)	16	25	0.64	5	23	0.22	0.013 *	21	48	0.44
SPD (m)	19	33	0.58	2	15	0.13	0.013 *	21	48	0.44
RS (m)	18	32	0.56	3	16	0.19	0.034 *	21	48	0.44

m: meters; Total distance (TD): High load (weeks with TD ≥ 21,900 m); Low load (weeks with TD < 21,900 m); High-speed distance (HSD): High load (weeks with HSD ≥ 288 m); Low load (weeks with HSD < 288 m); Sprint distance (SPD): High load (weeks with SPD ≥ 1601 m); Low load (weeks with SPD < 1601 m); Repeated sprints (RS): High load (RS ≥ 60). Low load (RS < 60); M: mean; *p* (*p* value); * (*p* < 0.05).

**Table 3:** Injury risk related to different load levels and sprint variables with OR and RR (mean).

Variables	High Load		Low Load		OR	CI 95%		RR	CI 95%	
	Injury (No Injury)	Total	Injury (No Injury)	Total		Min	Max		Min	Max
TD (m)	13 (10)	23	6 (19)	25	4.1	1.2	14.1	2.4	1.1	5.2
HSD (m)	14 (11)	25	5 (18)	23	4.6	1.3	16.3	2.6	1.1	6.0
SPD (m)	17 (16)	33	2 (13)	15	6.9	1.3	35.5	3.7	1.0	14.6
RS (m)	16 (16)	32	3 (13)	16	4.3	1.0	18.2	2.7	0.9	7.8

m: meters; n: numbers; Total distance (TD): High load (weeks with TD ≥ 21900 m); Low load (weeks with TD < 21900 m); High-speed distance (HSD): High load (weeks with HSD ≥ 288 m); Low load (weeks with HSD < 288 m); Sprint distance (SPD): High load (weeks with SPD ≥ 1601 m); Low load (weeks with SPD < 1601 m); Repeated sprints (RS): High load (RS ≥ 60). Low load (RS < 60); Injury (weeks with injuries). No injury (weeks without injuries). Total (total weeks). OR (Odds Ratio) RR (relative risk). CI95% (confidence interval); Min (minimum); Max (maximum).

**Table 4:** Relationship between injuries and different levels of load to find the expected time until new injuries (mean).

Variables	High Load		Low Load		*p*	Rate Ratio	CI 95%	
	λ	Expected Time Injury	λ	Expected Time Injury			Min	Max
TD (m)	0.65	1.5	0.24	4.2	0.046 *	2.7	1.0	8.6
HSD (m)	0.64	1.6	0.22	4.6	0.029 *	2.9	1.0	10.3
SPD (m)	0.58	1.7	0.13	7.7	0.033 *	4.3	1.0	38.2
RS (m)	0.56	1.8	0.19	5.3	0.067	3.0	0.9	15.9

Poisson: m: meters; n: numbers; Total distance (TD): High load (weeks with TD ≥ 21,900 m); Low load (weeks with TD < 21,900 m); High-speed distance (HSD): High load (weeks with HSD ≥ 288 m); Low load (weeks with HSD < 288 m); Sprint distance (SPD): High load (weeks with SPD ≥ 1601 m); Low load (weeks with SPD < 1601 m); Repeated sprints (RS): High load (RS ≥ 60). Low load (RS < 60); *p* (*p* value); * *p* < 0.05; CI 95% (Confidence interval); MIN (minimum); MAX (maximum); Rate ratio (Expected time injury low load/Expected time injury high load).

## Data Availability

The datasets used and/or analyzed during the current study are available from the corresponding author on reasonable request.
